# Poly Lactic-Co-Glycolic Acid- (PLGA-) Loaded Nanoformulation of Cisplatin as a Therapeutic Approach for Breast Cancers

**DOI:** 10.1155/2021/5834418

**Published:** 2021-06-28

**Authors:** Saad Alkahtani, Saud Alarifi, Gadah Albasher, Mohammed Al-Zharani, Nada H. Aljarba, Mohammed H. Almarzoug, Norah M. Alhoshani, Norah S. AL-Johani, Hani Alothaid, Abdullah A. Alkahtane

**Affiliations:** ^1^Department of Zoology, College of Science, King Saud University, Riyadh, Saudi Arabia; ^2^Department of Biology, College of Science, Imam Muhammad Ibn Saud Islamic University, Riyadh-11432, Saudi Arabia; ^3^Department of Biology, College of Sciences, Princess Nourah Bint Abdulrahman University, Riyadh, Saudi Arabia; ^4^Department of Basic Sciences, Faculty of Applied Medical Sciences, Al-Baha University, Al-Baha-65431, Saudi Arabia

## Abstract

Despite recent advancements in cisplatin (*cis*-diamminedichloroplatinum II) and other platinum-based chemotherapeutic drugs for treating solid tumors, their uses are limited by either in terms of toxicity and/or acquired drug resistance. These side effects have a dangerous problem with higher dose for severe patients. To overcome the low therapeutic ratio of the free drug, a polymeric nanoparticle drug delivery system has been explored promoting delivery of cisplatin to tumors. Recently, the applications of nanoparticles (NPs) have been underlined for encouraging the effects of chemotherapeutic drugs in cancerous cells. The intention of this project is to assess the potential of poly lactic-co-glycolic acid (PLGA) nanoparticles (NPs) for enhancing the effects of anticancer drug cisplatin. For the purpose, we have synthesized PLGA-cisplatin nanoparticles for increasing its bioavailability and studied the comparative cytotoxicity of free cisplatin and PLGA-cisplatin against MCF-7 cancer cell lines and HEK-293 normal cell lines. We have also analyzed the hallmarks of PLGA-cisplatin-induced apoptosis. The outcomes of this study may provide the possibility of delivery of anticancer drug to their specific site, which could minimize toxicity and optimize the drug efficacy.

## 1. Introduction

Cancer has exceeded cardiovascular and cerebrovascular diseases as the foremost cause of death worldwide [1–3]. Thus, the overload of cancer on healthcare systems consistently increases which requires more investigations to further advance an early and rapid recognition of such deadly disease [4, 5]. In particular, breast cancer (BC) is the common diagnosed noncutaneous form of solid tumor among women which is related with larger tumor size, higher grade, and frequent nodal immersion and becomes primary cause of death among women [6–8]. Although, the risk of BC consistently increases with different age groups, due to an elevated level of estrogen metabolite which generates reactive oxygen species (ROS) and prompts uncontrolled DNA production [9]. The incidence of BC is linked to numerous factors, among which the utmost common being its heterogeneous nature [10]. The inter/intratumoral heterogeneity, typically affecting the physical appearance of the breast and molecular variety, shows a pivotal role in its histology and staging [11]. The molecular stratification of BC is primarily based on the expression of hormonal receptors, namely, the estrogen receptor (ER) and progesterone receptor (PR), along with human epidermal growth factor receptor 2 (HER2) [10, 12, 13]. Owing to its heterogeneity, the treatment is complicated and the healing methodologies should be taken carefully [13].

In the last few decades, successful invention for cancer therapy is the use of metal-based pharmaceuticals after the serendipitous discovery of *cis*-Pt (NH_3_)_2_Cl_2_ has been extensively studied and remains a front-line treatment against a variety of solid tumors [14–16]. It was regularly accepted that cisplatin is a model chemotherapeutic drug of high proficiency and induces cytotoxicity by interference of transcription and/or the DNA replication mechanisms [17]. The interaction of the cisplatin drug and DNA forming DNA adducts leads to the activation of numerous signal transduction corridors involved in apoptosis [18]. However, there are certain limitations *viz*. severe toxicity, intrinsic and extrinsic resistance, and patient compliance [19, 20]. Additionally, cisplatin retains itself in tumor cell for a short duration due to low molecular weight and it can easily pass into the blood circulation causing damage to normal cells [21]. To conquer such issues, new strategies in the form of combination therapies were employed to improve the drug delivery profile of cisplatin towards the cancer cell line [22, 23]. It provides the possibility of targeted delivery of a certain anticancer drug to the tumor site, which could minimize toxicity and optimize the drug efficacy [24].

To date, various nanocarrier-based delivery systems like liposomes, micelles, and dendrimers have been taken that permit more directed release of the drugs [25–27]. These nanoparticles modify the drug release profile for the purpose of improving drug bioavailability because of their ability for tunable payload release to the specific target domain with minimum toxicity to surrounding healthy tissues. Among numerous biodegradable polymers, poly lactic-co-glycolic acid (PLGA) has been extensively used as drug carriers in clinical medicines as it is an FDA-approved copolymer [24, 28]. PLGA has a promising degradation characteristic that makes it ideal for sustained release for hydrophilic or hydrophobic drug. Furthermore, they can easily conjugate with specific target molecules and have the potential to alter their surface properties and improve interactions to reach specific tissues or cells [29]. Recently, Domínguez-Ríos et al. analyzed the significant improvement in the cytotoxicity along with an enhanced internalization of NPs as compared to free cisplatin for HER2 targeted ovarian cancer [30]. Similarly, Moreno et al. evaluated the *in vivo* efficacy of cisplatin-loaded PLGA nanoparticles administered to tumor-bearing mice, which provide a promising carrier for cisplatin avoiding its side effects without a decline of the efficacy [31].

**Figure 1:**
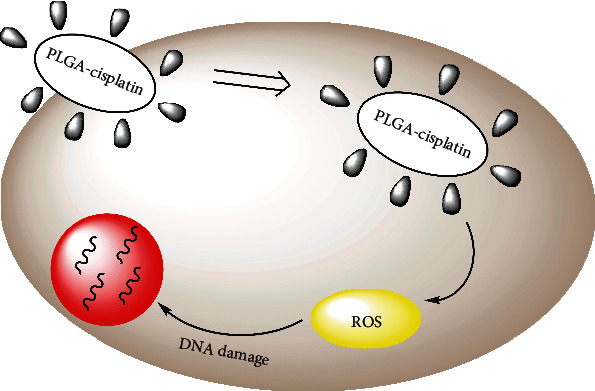
Schematic representation of encapsulation of PLGA-cisplatin NPs and their mode of action.

With this objective to treat breast cancer by utilizing the advantages of PLGA in this study, we chose PLGA nanoparticles as a core for the encapsulation of a cisplatin drug (Figure 1), for the purpose of safe delivery to the target-specific region. *In vitro* anticancer efficacy and cellular localization of this formulation were investigated on MCF-7 cancer cell lines. We assumed that delivery of drug would result in enhanced anticancer efficacy by modulating the tumor microenvironment to increase the penetration of PLGA NPs into the tumor cell.

## 2. Materials and Methods

### 2.1. Chemicals

Poly(D, L-lactide-co-glycolide) (PLGA, Resomer® RG 502 H Mw 7–17 kDa lactide: glycolide 50 : 50, acid terminated), cisplatin, dimethylformamide (DMF), polyvinyl alcohol (PVA, 9–10 kDa, 80% hydrolyzed), acetic acid, orthophenylenediamine (OPDA), phosphate buffer saline (PBS), 2-(N-morpholino) ethanesulphonic acid clorhydrate (MES) were purchased from Sigma-Aldrich. Constituents for cell culture media consist of fetal bovine serum (FBS), Dulbecco's modified Eagle medium (DMEM), trypsin, penicillin-streptomycin-neomycin (PSN) antibiotic cocktail, and ethylenediaminetetraacetic acid (EDTA) were procured from Gibco (Grand Island, NY, USA). Antibodies were bought from Santa Cruz Biotechnology, Dallas, Texas, USA, and eBioscience, San Diego, USA. Dyes were acquired from Thermo Fisher Scientific, USA. All other chemicals and solvents (purchased from Sigma-Aldrich) were used as received without further treatment.

### 2.2. Synthesis of PLGA-Cisplatin NPs

Cisplatin-loaded NPs were obtained by a nanoprecipitation technique in which 27 mg of PLGA was dissolved in minimum amount of DMF (2 mL) by vortexing. To this PLGA solution, cisplatin (10.5 mg) was added. Then, this organic solution was added dropwise to of PVA (20 mL) earlier dissolved in DI water (1% w/v) and stirred for few minutes. The excess amount of PVA and DMF were removed by centrifugation at 10,000 rpm (Thermo Sorvall Legend X1R) for 30 min at 20°C. The final pellet was dispersed in water (8 mL) followed by vortexing at 4°C and kept until apply within 1 h or lyophilized and stored for extended periods of time [31].

### 2.3. Fourier Transform Infrared (FTIR) Spectroscopy

The FTIR spectroscopy measurements were recorded to characterize the probable participation of the functional groups of cisplatin in synthesized PLGA-cisplatin. The FTIR spectra of PLGA-cisplatin NPs were acquired in an Avatar 330 FTIR spectrometer (Thermo Fisher Scientific, Waltham, MA, USA) over a scale of 4,250–500 cm^−1^ after adding with KBr pellets [32].

### 2.4. Atomic Force Microscopy (AFM)

To observe the surface morphology and measure the size of the resultant PLGA-cisplatin NPs, an AFM was applied. The sample was dropped onto newly cleaved mica slices and dried overnight. An AGILENT-N9445A series 5500 AFM (Agilent Technologies, Santa Clara, CA, USA), which was equipped with Pico View software, was used.

### 2.5. Cell Culture

The human embryonic kidney cells (HEK293) and breast cancer cell line (MCF-7) were achieved was acquired from ATCC, USA. These cells were culture in DMEM having 10% FBS with 1% antibiotic cocktail in a humidified medium under constant 5% CO_2_ at 37°C. Cell seeding was completed with EDTA (0.52 mM) and trypsin (0.25%) in phosphate-buffered saline (PBS) after 70−75% confluence. Later, the cells were plated at an essential concentration to allow them to reequilibrate before performing the experiment [33].

### 2.6. Determination of Cell Viability

Determination of cell viability was done by MTT [(4,5- dimethyl-thiazol-2-yl)-2,5-diphenyl-tetrazolium bromide] assay [34]. HEK293 and MCF-7 cells were seeded at a required density (4 × 10^3^ cells/well) in a 96-well plate from the respective plate. After 18−24 h of seeding, cells were treated with cisplatin and PLGA-cisplatin at 0-50 (*μ*g/mL) followed by an initial screening for different time duration. After treatment, plates were placed for 24 h in an incubator at 37°C in a humidified CO_2_-rich condition (5%). After incubation for 24 h, cells were rinsed from each well of 96-well plates by PBS. Then, the MTT solution was added to each well and reserved in an incubator for 4 h to appear formazan dye. The formazan dye was then solubilized using DMSO, and the absorbance was taken at 595 nm using an ELISA reader (EMax, Molecular Device, USA) [35]. Cell propagation was evaluated from the absorption intensity in terms of cell viability as follows:

(1) $$\begin{array}{c} \text{Cell viability}=\frac{\text{OD of Control}-\text{OD of treated}}{\text{OD of Control}}\times 100. \end{array}$$

In each case, the PLGA-cisplatin was sonicated before considering in a cell line to obtain homogenized mixtures. Each experiment was repeated thrice to obtain reported biological results.

### 2.7. Detection of Apoptosis/Necrosis

Necrotic and apoptotic cell death was determined using the Annexin-V FITC/PI apoptosis detection kit (Calbiochem, CA, USA). The MCF-7 cells were plated in six-well plates then treated with PLGA-cisplatin at 23 *μ*g/mL. After 12 and 24 h of incubation, the cells were washed and stained with propidium iodide (PI) and Annexin-V-FITC in the presence of binding buffer as per the manufacturer's protocols [36]. The early and late apoptotic percentages, live, and necrotic cells were analyzed using a flow cytometer (BD LSRFortessa™ San Jose, CA, USA). The acquired data were analyzed using FlowJo (version 10.0) software.

### 2.8. DNA Fragmentation Assay

Treated cells with the PLGA-cisplatin nanoparticles at different concentrations (0-40 *μ*g/mL) were analyzed for 24 h. Then, the resultant fragmented DNA was calculated using commercially obtainable kits at 405 nm according to the manufacturer's procedure [37].

### 2.9. Assessment of Intracellular ROS

Intracellular mitochondrial ROS have been calculated following the previously reported protocols [38]. To adopt the intercellular ROS, we incubated the treated MCF-7 cells with 10 *μ*M 2′,7′-dichlorofluorescein diacetate (DCF-DA) for 25 min. The slides were then counterstained with DAPI for 10 min and mounted with the ProLong Antifade Reagent (Molecular Probe, Eugene, OR, USA). Later, the slides were subjected to confocal laser scanning microscope (FV 10i, Olympus, Japan) [39]. The change of DCF-DA fluorescence used as a fluorescent indicator of ROS formation in cell culture-based antioxidant assays.

### 2.10. In Vitro Cisplatin Release

The *in vitro* cisplatin release experiments were conducted at pH 5.5 with 10 *μ*g/mL of cisplatin containing PLGA. 1 mL of PLGA-cisplatin was loaded into a dialysis bag, then dispersed in a 50 mL of PBS of pH 7.4 at 37°C. Further, the full system was transferred at 130 rpm for 24 h. From the above dispersions after 2 h intervals, 500 *μ*L aliquots were drawn and it was replaced by an equal volume of the fresh PBS solution for retaining the same release medium. The released amount of cisplatin was determined spectrophotometrically at 510 nm [40].

### 2.11. Statistical Analysis

Data were represented as mean ± SEM of the multiple data points. Statistical significance in the deference was calculated using analysis of variance (ANOVA) using OriginPro (version 8.0) software where *p* < 0.05 was considered significant.

## 3. Result and Discussion

### 3.1. Characterization of PLGA-Cisplatin NPs

The encapsulation of weakly soluble and toxic drugs within polymeric NPs increases their therapeutic and pharmacodynamics performance and decreases their side effects [41]. Cisplatin is poor soluble in water (0.23 g/100 mL of water) and organic solvents makes it quite difficult to prepare concentrated solutions necessary to produce NPs. Although DMSO is well known to be a good solvent for cisplatin, it has been reported to compromise the cytotoxic potency of the drug, so DMF was chosen as an organic solvent for the preparation of NPs.

**Figure 2:**
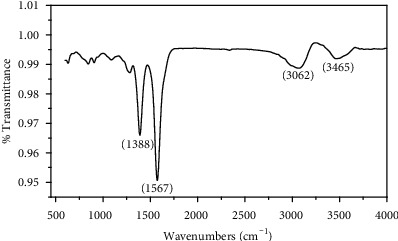
FTIR data of PLGA-cisplatin.

The FTIR spectrum of the sample after the encapsulation of cisplatin is reported (Figure 2). Two peaks were found in the 3062–3465 cm^−1^ (related to asymmetric and symmetric stretching of -NH group) and 1600–1300 cm^−1^ (related to HNH asymmetric and symmetric bending). Some peaks were observed in between 500 and 1300 cm^−1^ (viz. at 956 cm^−1^ for –OH bending), which are the characteristic peaks of PLGA.

**Figure 3:**
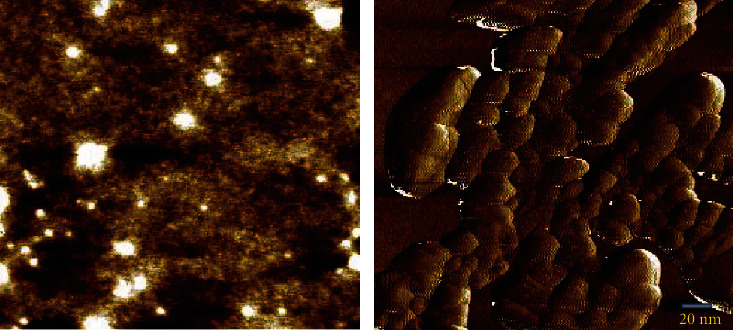
AFM data of PLGA-cisplatin.

Then, we have studied the size and shape of the PLGA-cisplatin NPs by AFM. The AFM data (Figure 3) suggest that PLGA-cisplatin exhibits nanosphere-like shape. We have calculated the size which is around 95.56 ± 7.94 nm from the AFM images.

### 3.2. PLGA-Cisplatin NPs Induced Cytotoxicity

**Figure 4:**
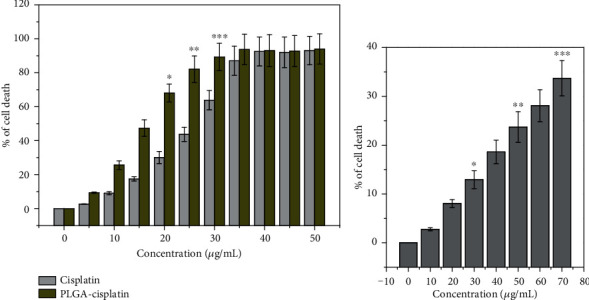
(a) Rate of cell death of MCF-7 cells after treatment with cisplatin only and PLGA-cisplatin for 24 h as evaluated by MTT assay. (b) Rate of cell death of HEK-293 cells after treatment with PLGA-cisplatin NPs. Each value represents the mean ± SE of three experiments. *n* = 3 (^∗^*p* < 0.05, ^∗∗^*p* < 0.01, and ^∗∗∗^*p* < 0.001), compared with the control.

The cytotoxic effects of PLGA-cisplatin NPs have been tested against the breast cancer cell line (MCF-7) by MTT assay. Based on the values of IC_50_, different concentrations between 0 and 50 *μ*g/mL of PLGA-cisplatin NPs were selected and the treatment period was for a 24 h period. The MTT assay data cleared that the cell death has been increased with the concentration of PLGA-cisplatin NPs. We have also evaluated the cytotoxicity of cisplatin in the same concentration range. From the MTT assay, we have acquired the IC_50_ value of PLGA-cisplatin NPs is 23 ± 2.80 *μ*g/mL, whereas in case of only cisplatin, the IC_50_ value is 38 ± 3.78 *μ*g/mL. This MTT assay data cleared that PLGA-cisplatin NPs are superior cytotoxic than cisplatin only (Figure 4(a)). We have also checked the cytotoxicity of NPs in the HEK-293 cell line. The data concluded that, after 40 *μ*g/mL, significant cell death was found (Figure 4(b)).

### 3.3. PLGA-Cisplatin NPs Induced Apoptosis in MCF-7 Cells

**Figure 5:**
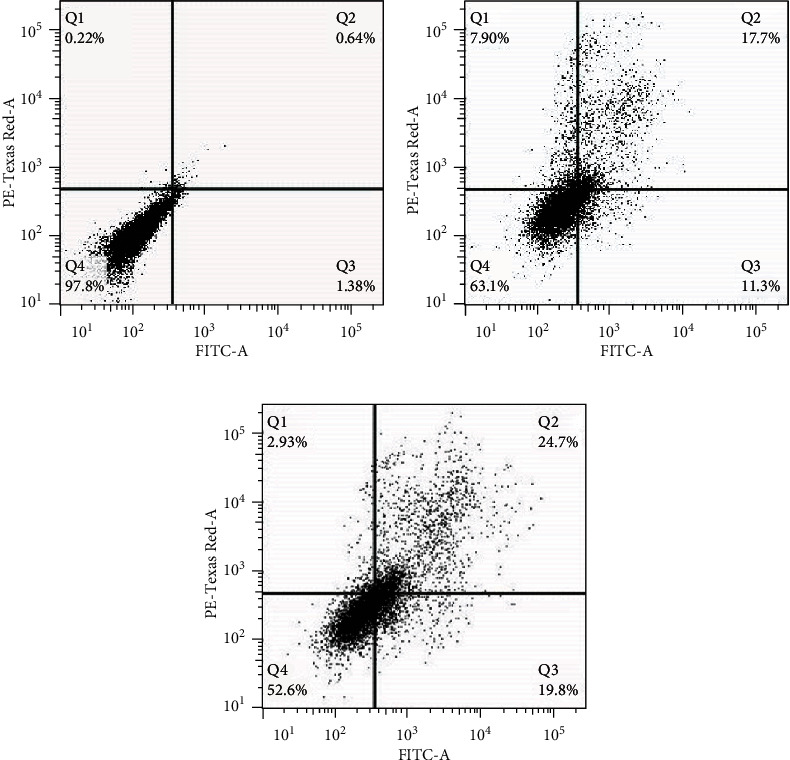
Annexin-V/PI staining to analyze apoptosis/necrosis in MCF-7 cells. Time-dependent study (12 and 24 h) after treatment of cells with 23 *μ*g/mL of PLGA-cisplatin. (a) Untreated cells. (b) Differences in the percentage of cell death in the treated cells for 12 h. (c) Differences in the percentage of cell death in treated cells for 24 h.

To examine whether the PLGA-cisplatin nanosphere was involved in apoptosis/necrosis, flow cytometric assessment was performed using Annexin-V-FITC/PI staining by analyzing the elevated level of serine phosphatidyl in the outer membrane of cells. As shown in (Figure 5), it was evident that the percentages of apoptotic (early and late) cells have been enhanced in a time-dependent manner (11.3% EA/17.7% LA for 12 h and 19.8% EA/24.7% in LA for 24 h) after treatment of 23 *μ*g/mL of PLGA-cisplatin NPs, with respect to the control cells (1.38% EA and 0.64% LA). These results suggested that PLGA-cisplatin NPs-induced cell death was directly correlated with cytotoxicity followed by apoptosis.

### 3.4. Assessment of Intracellular ROS Generation

**Figure 6:**
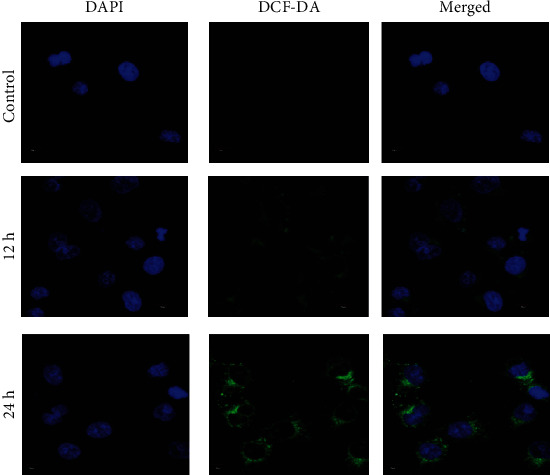
Measurement of ROS generation after a time-dependent treatment (12 and 24 h) of 23 *μ*g/mL PLGA-cisplatin (DAPI-nuclear stainer).

Cisplatin is a well-known ROS inducer [42]. So, we have established the ROS generation by confocal microscopy after PLGA-cisplatin NP treatment (23 *μ*g/mL) in a time-dependent manner (12 and 24 h) using the DCF-DA dye as an indicator of ROS. Increase in DCF-DA fluorescence emission (595 nm) is the indication of increase in ROS (Figure 6). Here, DAPI has been taken as a nuclear stainer. As shown in Figure 5, the microscopic images showed that the green emission has been increased in a time-dependent manner after treatment of 23 *μ*g/mL of PLGA-cisplatin NPs. This above data confirmed that PLGA-cisplatin NPs are increasing ROS in theMCF-7 cell line.

### 3.5. Detection of DNA Damage

**Figure 7:**
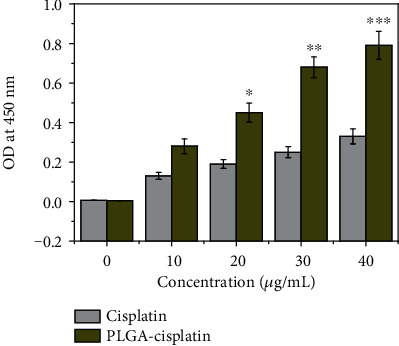
DNA fragmentation assay: cisplatin- and PLGA-cisplatin- (0-40 *μ*g/mL) treated MCF-7 cells. Each value represents the mean ± SE of three experiments, *n* = 3 (^∗^*p* < 0.05, ^∗∗^*p* < 0.01, and ^∗∗∗^*p* < 0.001), compared with control.

ROS overload also prompts oxidative damage to the cell's biomacromolecules, which culminates in the cell dysfunction and death. At high levels, ROS can lead to impaired physiological function through cellular damage of DNA. Hence, the DNA fragmentation assay was accomplished for both cisplatin and PLGA-cisplatin NPs, respectively, at 0-40 *μ*g/mL concentrations. For the purpose, we have selected the OD value after treatment at 405 nm (Figure 7). The OD value suggests that the DNA fragmentation has been increased with the concentration of cisplatin and PLGA-cisplatin NPs. However, in case of PLGA-cisplatin NPs, the DNA fragmentation was found to be higher. This observation cleared that the elevated amount of ROS is damaging the DNA. These data showed that ROS-mediated DNA damage is correlated with PLGA-cisplatin NP-induced apoptosis in MCF-7 cancer cells.

### 3.6. Drug Release of Cisplatin from PLGA-Cisplatin NPs

**Figure 8:**
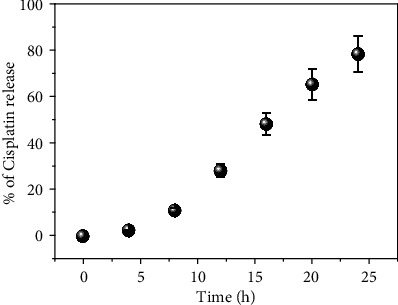
Time-dependent cisplatin release from PLGA-cisplatin at pH -5.5 (UV-Vis spectroscopic data). Each value represents the mean ± SE of three experiments (*n* = 3).

We have also checked the delivery of cisplatin from PLGA-cisplatin NPs at buffer of pH -5.5 by dialysis membrane technique. The measured the OD value at 4 h interval and extended up to further 24 h. It has been perceived that after 16 h almost 50% of cisplatin has been released, which confirmed that the bioavailability of PLGA-cisplatin NPs is more superior than only cisplatin (Figure 8).

## 4. Conclusions

In this study, cisplatin-loaded PLGA NPs possessing chemotherapy properties were obtained by a multistep process. PLGA-cisplatin NPs were characterized by AFM and FTIR techniques. The cytotoxicity data of the nanoparticles along with only cisplatin into MCF-7 have been evaluated by MTT Assay. A colorimetric UV–Vis method was adapted to quantify cisplatin in buffer samples. The pH-dependent drug release profile of NPs was observed on the surrounding medium, favoring the release of cisplatin under an acidic environment. In this way, we confirmed that the bioavailability of PLGA-cisplatin NPs has been increased. As in case of PLGA-cisplatin, the anticancer efficacy is higher compared to only cisplatin, so the risk of severe physiological disorders will be reduced. We have also established that the ROS generation into MCF-7 has been increased after PLGA-cisplatin nanoparticle treatment in a time-dependent manner. After that, we have checked the DNA damage which is following ROS generation data so we can say ROS generation is the cause of DNA damage in PLGA-cisplatin-induced apoptosis. Thus, PLGA-cisplatin NPs has huge potential to be employed as an anticancer agent to overcome the epidemiology of cancer in future.

## Data Availability

The data generated or analyzed in this article are publicly available online without request.

## Abbreviations

PLGA: Poly lactic-co-glycolic acid
BC: Breast cancer
ROS: Reactive oxygen species
ER: Estrogen receptor
PR: Progesterone receptor;
HER2: Human epidermal growth factor receptor
DMF: Dimethylformamide
PVA: Polyvinyl alcohol
OPDA: Orthophenylenediamine
OD: Optical density
MES: Ethanesulphonic acid clorhydrate
FTIR: Fourier transforms infrared
AFM: Atomic force microscopy
PSN: Penicillin-streptomycin-neomycin
DCF-DA: 2′,7′-Dichlorofluorescein diacetate.
